# Effects of pulmonary rehabilitation combined with inspiratory muscle training on lung function and exercise capacity in older patients with COPD: a systematic review and meta-analysis

**DOI:** 10.3389/fmed.2025.1621375

**Published:** 2025-07-09

**Authors:** Jun Xie, Yang Zhu, Ya Wang, Yunjun Mo, Xiaohui Shi, Wen-Ming Liang, Fei-Fei Ren, Zhenmin Bai, Feng Nie

**Affiliations:** ^1^Department of Rehabilitation Medicine, Chengdu Seventh People’s Hospital, Affiliated Cancer Hospital of Chengdu Medical College, Chengdu, China; ^2^School of Sports Medicine and Rehabilitation, Beijing Sport University, Beijing, China; ^3^BaoLin Central Health Center, Ziyang, China; ^4^Physical Education Institute, Jimei University, Xiamen, China; ^5^Department of Physical Education, Beijing Language and Culture University, Beijing, China

**Keywords:** inspiratory muscle training, COPD, pulmonary function, exercise capacity, pulmonary rehabilitation

## Abstract

**Background:**

Pulmonary rehabilitation is central to COPD management, with inspiratory muscle training (IMT) as a key component. However, evidence is inconsistent on whether combining PR with IMT offers added benefits for older COPD patients.

**Objective:**

To evaluate the comparative effects of PR combined with IMT versus PR alone on key outcomes in older COPD patients, including quality of life [St. George’s Respiratory Questionnaire (SGRQ)], exercise tolerance [6-min walk distance (6MWD)], respiratory muscle strength [maximal inspiratory pressure (PImax)], and pulmonary function metrics (FEV_1_, FEV_1_%).

**Methods:**

A systematic search of PubMed, EMBASE, Web of Science, and the Cochrane Library (January 2005–January 2025) identified randomized controlled trials (RCTs) meeting criteria: (1) participants were ≥ 55 years old with GOLD stage II–IV COPD; (2) interventions compared PR combined with IMT versus PR alone; (3) outcomes included PImax, FEV_1_, FEV_1_%, SGRQ, and 6MWD. Non-English and animal studies were excluded. Risk of bias was assessed using Cochrane RoB 2.0, and the certainty of evidence was evaluated via the GRADEpro 3.6.1.

**Results:**

Nine RCTs (582 patients) were included. Compared with PR alone, PR combined with IMT did not improve 6MWD (SMD = 0.15, 95% CI: −0.11–0.42; low-quality evidence) or SGRQ scores (SMD = −0.19, 95% CI: −0.38–0.01, low-quality evidence). PImax improved moderately (SMD = 0.78, 95% CI: 0.44–1.13, I^2^ = 48.7%, low-quality evidence). FEV_1_ and FEV_1_% trended upward (SMD = 0.50 and 0.58, respectively) but showed high heterogeneity (FEV_1_: I^2^ = 72.9%, *p* = 0.025, very low-quality evidence; FEV_1_%: I^2^ = 75.6%, *p* = 0.006, low-quality evidence), precluding significance. Subgroup analyses showed significant PImax improvements in interventions lasting ≥ 12 weeks (SMD = 0.866, 95% CI: 0.579–1.153; I^2^ = 0%) or with weekly cumulative durations ≥ 180 min (SMD = 0.922, 95% CI: 0.666–1.177; I^2^ = 0%), with no 6MWD benefits in any subgroup.

**Conclusion:**

Low-quality evidence indicates that PR combined with IMT improves respiratory muscle strength (PImax) in older COPD patients versus PR alone, with no significant benefit for exercise capacity (6MWD) or lung function. For older COPD patients, ≥ 12-week PR combined with IMT interventions (sessions > 60 min; weekly duration ≥ 180 min) may enhance PImax.

**Systematic review registration:**

https://www.crd.york.ac.uk/PROSPERO/view/CRD420251010168, CRD420251010168.

## 1 Introduction

Chronic obstructive pulmonary disease is a respiratory disease characterized by persistent airflow limitation and progressive decline in lung function. The World Health Organization (WHO) predicts that COPD will become the third leading cause of death worldwide by 2030, with its disease burden being particularly pronounced in older populations (1). A large-scale epidemiological study in China reported a COPD prevalence of 13.7% among adults aged 40 years and older (2). In older COPD patients, reduced lung elasticity and accelerated alveolar structural damage lead to an accelerated decline in forced expiratory volume in 1 s (FEV_1_), with an annual decline rate approximately 2.5-fold higher than that in healthy individuals (3). This significantly exacerbates the decline in exercise endurance and deterioration of quality of life.

Current clinical management of COPD centers on medications such as bronchodilators (4). However, these drugs have limited efficacy in improving lung function (FEV_1_/FEV_1_%) and cannot reverse respiratory muscle weakness (e.g., maximal inspiratory pressure (PImax) < 60 cmH_2_O) or declining exercise endurance (5). Pulmonary rehabilitation (PR), a non-pharmacological intervention, improves functional status through physical activities such as aerobic exercise and resistance training (Report). Multiple studies demonstrate that exercise-based PR enhances exercise capacity, quality of life, and reduces dyspnea more effectively than non-exercise programs in COPD patients (6–9). Consequently, PR is recognized as the most cost-effective therapeutic strategy (10). Inspiratory muscle training (IMT), a key component of PR, significantly improves respiratory function and exercise capacity in COPD patients (11–13). A report by the American Thoracic Society (ATS) suggests that IMT, used as an independent intervention or added to PR for patients with respiratory muscle weakness, may offer benefits (10). However, existing systematic reviews are inconsistent regarding the efficacy of PR combined with IMT: Cochrane reviews indicate that the superiority of PR combined with IMT versus PR alone in improving dyspnea, functional exercise capacity, and quality of life remains unclear (14–16).

This study aims to systematically compare the intervention effects of PR combined with IMT versus PR on quality of life [St. George’s Respiratory Questionnaire (SGRQ)], 6-min walk distance (6MWD), respiratory muscle strength (PImax), and lung function (forced expiratory volume in 1 s (FEV_1_), forced expiratory volume in 1 s% predicted (FEV_1_%)) in older COPD patients through meta-analysis. Subgroup analyses will explore how intervention parameters (e.g., frequency, duration) influence treatment efficacy, providing evidence-based insights for developing personalized non-pharmacological intervention protocols for elderly COPD patients.

## 2 Data and methods

This study adhered to the Preferred Reporting Items for Systematic Reviews and Meta-analyses (PRISMA 2020) guidelines (17) and was registered on PROSPERO (CRD420251010168).

### 2.1 Search strategy

We searched PubMed, EMBASE, Web of Science, and the Cochrane Library for randomized controlled trials (RCTs) evaluating the effects of exercise on lung function and exercise capacity in older COPD patients from January 2005 to January 2025. We also manually searched the reference lists of relevant studies, such as reviews and meta-analyses, to identify additional related studies. Two authors (JX and YW) independently conducted the search process, and any disagreements were resolved through discussions with the third author (YZ).

### 2.2 Inclusion and exclusion criteria

This study applied the PICO framework to define inclusion criteria as follows (18) (Supplementary Table 1): ① Population: Patients aged ≥ 55 years, diagnosed with stable GOLD (19) stages II–IV according to GOLD criteria; ② Intervention: Evidence-based IMT protocols (e.g., threshold loading, diaphragmatic electrical stimulation) combined with comprehensive PR strategies, including exercise training, nutritional support, and psychological interventions; ③ Comparison: Standardized PR strategies implemented as single interventions (i.e., without additional IMT); ④ Outcome: Primary outcomes included: Maximum inspiratory pressure (PImax, cmH_2_O); Pulmonary function metrics: FEV_1_ and FEV_1_% predicted; SGRQ score; 6MWD. ⑤ Study Design: Randomized controlled trials (RCTs) only.

Exclusion Criteria: ① non-RCT publications (e.g., conference abstracts, review articles, case reports, observational studies, non-peer-reviewed manuscripts); ② animal or preclinical studies; ③ articles not published in English.

### 2.3 Data extraction

Data extraction was independently conducted by two authors (JX and YW), with all extracted information cross-checked for consistency and discrepancies resolved through discussion with a third author (YZ). The extracted details included basic study characteristics such as the first author’s surname and initials, publication year, and country/region of the study; participant characteristics comprising sex (male/female), age, sample size, and GOLD stage; intervention specifics including types of respiratory muscle training (e.g., threshold loading, inspiratory muscle training) and pulmonary rehabilitation strategies (e.g., exercise protocols, nutritional support), along with session duration (minutes per session), frequency (sessions per week), weekly cumulative duration (total minutes per week), and intervention duration (total weeks); and outcome measures such as PImax, FEV_1_, FEV_1_% predicted, SGRQ scores, and 6MWD results reported as mean ± standard deviation.

### 2.4 Methodological quality assessment

The methodological quality of the included studies was independently assessed by two authors (JX and YW) using the Cochrane Risk of Bias 2.0 (RoB 2.0) tool (20). Discrepancies in risk assessment were resolved through discussions with a third author (YZ) to ensure consensus. The RoB 2.0 tool evaluates bias across five domains: randomization process, deviations from assigned interventions, missing outcome data, measurement of the outcome, and selection of the reported result. Each domain was classified as low risk, high risk, or some concerns according to predefined criteria, thereby facilitating a rigorous evaluation of the methodological quality of the included studies. To further synthesize and evaluate the evidence certainty based on these quality assessments, we employed the GRADEpro tool (version 3.6.1).

### 2.5 Statistical analysis

Statistical analyses were performed using Stata 18.0 software. For continuous outcomes, effect sizes (ES) were calculated as standardized mean differences (SMDs) with 95% confidence intervals (CIs). When meta-analysis was precluded due to insufficient data, Hedges’ g was utilized to estimate ES magnitudes (21). Cohen’s conventional thresholds (SMD = 0.2, 0.5, and 0.8) were applied to interpret small, moderate, and large effects, respectively (22). Heterogeneity was assessed using the I^2^ statistic and Cochran’s Q-test (*p* < 0.10), with I^2^ values of 25%, 50%, and 75% indicating low, moderate, and high heterogeneity (23). A fixed-effects model was selected when I^2^ ≤ 50% and *p* ≥ 0.10; otherwise, a random-effects model was employed. Subgroup analyses stratified by intervention duration, frequency, session duration, and weekly cumulative duration to evaluate their impact on exercise capacity and lung function in older COPD patients. Meta-regression further examined associations between these intervention parameters (duration, frequency, session duration, weekly cumulative duration) and effect sizes. Sensitivity analyses, forest plots, funnel plots, and publication bias assessments (Egger’s test) were conducted to validate results robustness. Statistical significance was defined as *p* < 0.05.

## 3 Result

### 3.1 Inclusion results

**FIGURE 1:**
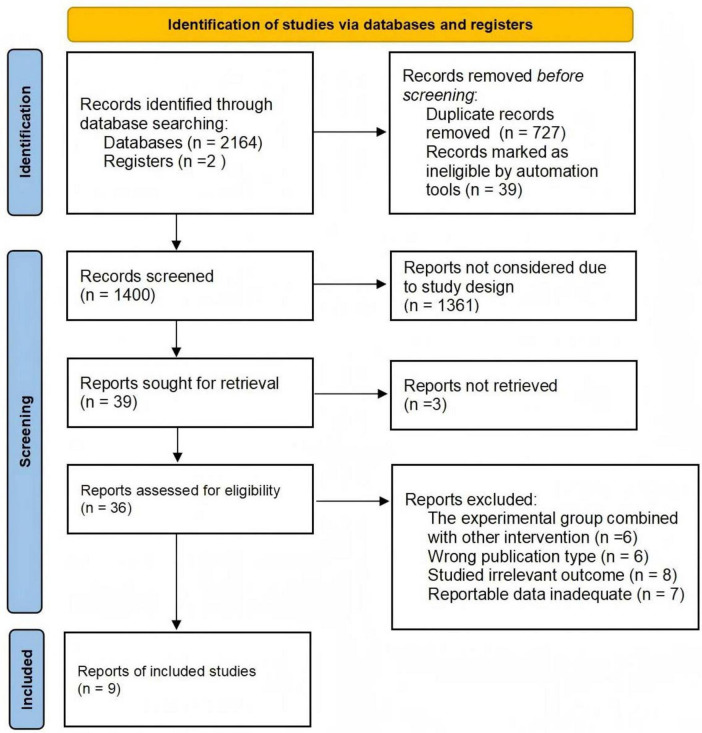
PRISMA flowchart of study selection.

A total of 2166 studies were identified from four databases (Figure 1). After excluding duplicates, 1400 studies were retained, and 36 studies remained after screening titles and abstracts. Twenty-seven studies were excluded for the following reasons: (1) The experimental group combined with other intervention (*n* = 6); (2) Wrong publication type (*n* = 6); (3) Studied irrelevant outcome (*n* = 8); (4) Reportable data inadequate (*n* = 7). Finally, 9 studies met the inclusion criteria (24–32).

### 3.2 Study characteristics

The baseline characteristics of the nine included RCTs are summarized in Table 1. All studies were published within the past two decades (24–32), enrolling a total of 582 participants, with 287 assigned to the PR combined with IMT group and 295 to the control alone group. Sample sizes ranged from 9 to 109 participants, and the mean age of participants was predominantly over 60 years, except for the PR combined with IMT group in Tout et al. (26), which reported a mean age under 60 years. Notably, older mean ages (approximately 70 years) were observed in the studies by Mador et al. (24) and Wang et al. (29). Regarding COPD severity, most participants were classified as GOLD stage II or higher: two studies included participants with GOLD stages II–III (26, 27), three studies enrolled patients across stages II–IV (29, 31, 32), and four studies focused exclusively on stages III–IV (24, 25, 28, 30). According to the COPD staging criteria based on FEV_1_ (33), the included studies covered severe cases (24–26, 28, 30, 31) and moderate cases (29). The two study only reported absolute FEV_1_ values (27, 32). According to the functional impairment classification based on PImax absolute values and negative value criteria (34), the included studies covered severe impairment (28), moderate impairment (31), and mild impairment (24, 25, 29, 30, 32). Two studies did not report baseline PImax values (26, 27).

**TABLE 1:** Baseline characteristics of included participants.

References	Country	Sample size (CG/EG)	Average age		GOLD stage	FEV_1_(L) FEV_1_%pred (%)	PImax (cmH_2_0)
			CG	EG			
(27)	United States of America	13/9	61.5 ± 6.1	62.3 ± 5.2	II, III	PR + IMT: 1.8 ± 0.7 PR: 1.3 ± 0.4	–
(32)	France	16/16	62.5 ± 5	63 ± 4	II, III, IV	IMT + PR: 1.70 ± 0.05 L PR: 1.70 ± 0.06 L	IMT + PR: 61.9 ± 21.8 PR: 64.9 ± 21.7
(30)	France	74/75	62.2 ± 8.0	65.9 ± 8.9	III, IV	IMT: 36.4% ± 9.5% IMT + PR: 34.2% ± 8.4%	IMT: 66.2 ± 21.7 IMT + PR: 64.8 ± 23.0
(31)	Belgium	110/109	66 ± 8	65 ± 7	II, III, IV	IMT + PR: 40% ± 15% Sham IMT + PR: 43% ± 17%	IMT + PR: 52 ± 14 Sham IMT + PR: 51 ± 12
(28)	Germany	15/14	66 ± 8	66 ± 7.5	III, IV	Sham IMT + PR: 31.8% ± 11.7% IMT: 35.7% ± 12.0%	Sham-IMT: −28.8 ± 10.4 IMT: −34.9 ± 7.9
(24)	United States of America	15/14	69.7 ± 7.7	70.9 ± 7.4	III, IV	PR: 1.44 ± 0.10 L (43.6% ± 3.5%) PR + IMT: 1.49 ± 0.16 L (45.1% ± 5.5%)	PR: 65.1 ± 6.8 PR + IMT: 65.4 ± 8.3
(25)	Israel	14/13	65.2 ± 13.6	66.1 ± 12.39	III, IV	PR + IMT: 1.28 ± 0.4 L (45% ± 2.4%) PR + Sham IMT: 1.29 ± 0.4 L (46% ± 2.7%)	PR + IMT: 66 ± 4.7 PR + Sham IMT: 67 ± 4.6
(26)	Lebanon	10/10	61 ± 9.32	58.1 ± 8.72	II, III	IMT + PR: 0.93 ± 0.39 L PR: 0.98 ± 0.32 L	–
(29)	China	28/27	70.8 ± 4.5	70.6 ± 6.3	II, III, IV	PR + IMT: 1.19 ± 0.4L (49.82% ± 16.14%) PR: 1.33 ± 0.47L (51.26% ± 18.00%)	PR + IM: 72.40 ± 20.41 PR: 74.66 ± 13.83

CG, control group; EG, experimental group; SGRQ, St. George’s Respiratory Questionnaire; 6MWD, 6-min walk distance; PImax, respiratory muscle strength; FEV_1_, forced expiratory volume in 1 s; FEV_1_%, forced expiratory volume in 1 s% predicted; PR, pulmonary rehabilitation; IMT, inspiratory muscle training.

Intervention durations varied (Table 2), with two studies implementing a 4-week protocol (28, 30), five studies adopting an 8-week intervention (24, 26, 27, 29, 32), and two studies utilizing longer durations of 12 weeks (31) and 24 weeks (25).

**TABLE 2:** Frequency and interventions of PR and IMT.

References	Control group		Experimental group		Indicators
	Intervention measures	Frequency	Intervention measures	Frequency	
(27)	Cycling, warm-up, and cool-down	PR: 8 weeks, 3 times/week, 23–45 min/time	1. IMT: threshold IMT 2. PR interventions versus the control group	IMT: 8 weeks, 5 times/week, 2 times/day, 5–15 min/time	SGRQ FEV_1_ FEV_1_%
(32)	Treadmill, upper and lower limb stretching.	ET: 8 weeks, 3 times/week, 30 min/time	1. IMT: power breathe medic 2. PR interventions versus the control	IMT: 8 weeks,7 times/week	PImax 6MWT
(30)	Aerobic exercise, upper and lower limb muscle strengthening training, therapeutic education programs, group aerobic gymnastics, smoking cessation programs, and psychosocial and dietary advice.	PR: 4 weeks, 5 days/week, 60 min/time	1. IMT: power breathe medic 2. PR interventions versus the control	IMT: 4 weeks, 5 days/week, 2 times/day, 15 min/time	SGRQ PImax 6MWD
(31)	1. PR: treadmill or bicycle, and upper and lower limb muscle training. 2. Sham IMT: power breathe KHP2	GET: 20 sessions (Germany)–36 sessions (other centers), with a frequency of 3–5 times/week, approximately 60 min/time	1. IMT: power breathe KHP2 2. PR interventions versus the control	IMT: 12 weeks, 7 times/week, 1 time/day	6MWD PImax
(28)	1. PR: dynamometer training, weight training 2. Sham IMT: Threshold IMT	PR: 4 weeks, 7 days/week, 2 times/day, 1 h/time	1. IMT: Respifit S trainer 2. PR interventions versus the control	IMT: 4 weeks, 5 times/week	6MWD Pimax FEV_1_ FEV_1_%
(24)	PR: dynamometer and treadmill.	PR: 8 weeks, 3 times/week, 60 min/time	1. IMT: breathing with a side-port breathing bag. 2. PR interventions versus the control	IMT: 8 weeks, 3 times/week, 15–20 min/time	Pimax 6MWD
(25)	1. PR: endurance exercise and resistance training. 2. IMT: sham IMT	PR: 24 weeks, 1 h/time, 3 times/week	1. IMT: power breathes 2. PR interventions versus the control	IMT: 24 weeks, 30 min/time, 3 times/week	PImax SGRQ 6MWT FEV_1_
(26)	PR: routine care and muscle training.	PR: 8 weeks, 2 times/week, 20–30 min/time	1. IMT: threshold1 IMT 2. PR interventions versus the control	IMT: 8 weeks, 2 times/week, 20–30 min/time	FEV_1_ SGRQ 6MWD
(29)	PR: bicycle	PR: 8 weeks, 3 times/week, 30 min/time	1. IMT: threshold-loaded IMT device 2. PR interventions versus the control	IMT: 8 weeks, 3 times/week, 14 min/time	PImax 6MWD FVC_1_ FEV_1_% SGRQ

PR, pulmonary rehabilitation; IMT, inspiratory muscle training; SGRQ, St. George’s Respiratory Questionnaire; 6MWD, 6-min walk distance; PImax, respiratory muscle strength; FEV_1_, forced expiratory volume in 1 s; FEV_1_%, forced expiratory volume in 1 s% predicted.

### 3.3 Risk of bias

**FIGURE 2:**
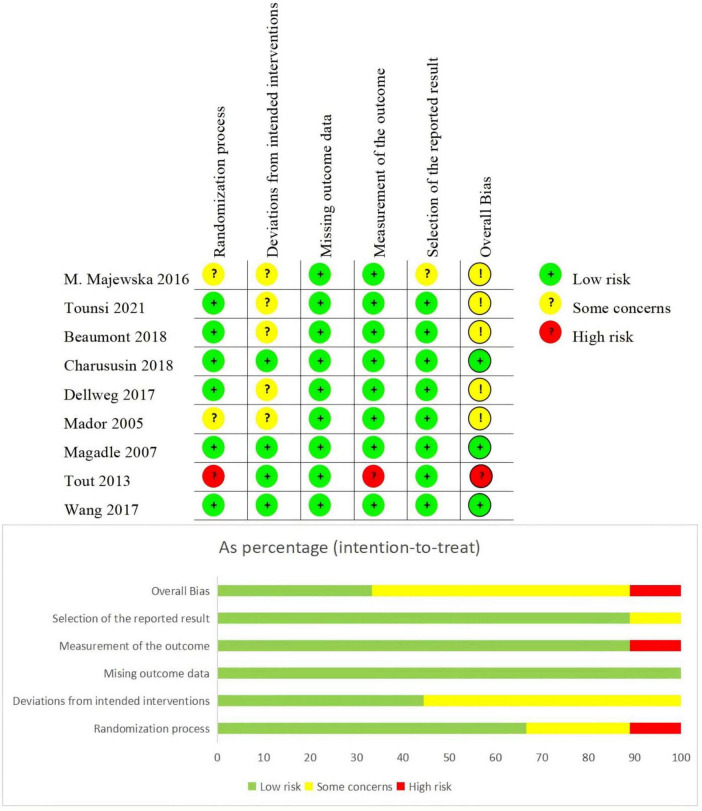
Bias risk assessment.

This study utilized the Cochrane RoB 2.0 tool to evaluate the methodological quality of included studies, assessing risks of selection bias, implementation bias, detection bias, attrition bias, reporting bias, and other biases (Figure 2). The bias risk assessment revealed that most of the 9 included studies demonstrated strong control over the randomization process (6/9 low risk), intervention implementation (all low risk with some procedural considerations in partial cases), and outcome measurement (8/9 low risk), indicating generally high methodological quality. Overall, 5 studies were rated as low risk of bias, 3 had partial methodological flaws, and 1 was deemed high risk.

### 3.4 Meta-analysis results

#### 3.4.1 Effect of PR combined with IMT on 6MWD in older COPD patients

**FIGURE 3:**
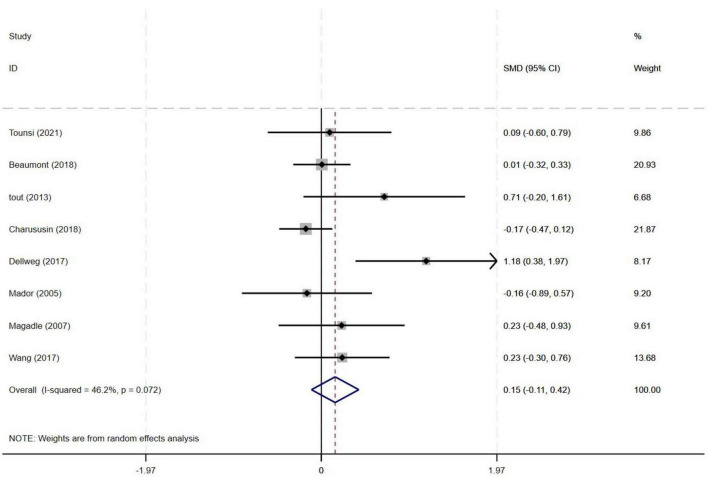
Effect of PR combined with IMT on 6MWD in older COPD patients.

Among the nine included studies, eight compared the effects of PR combined with IMT versus PR alone on the 6MWD in older COPD patients. The meta-analysis revealed a pooled SMD of 0.15 (95% CI: −0.11–0.42) for 6MWD, indicating no statistically significant difference between the PR combined with IMT and PR. Heterogeneity across studies was moderate (I^2^ = 46.2%, *p* = 0.072) (Figure 3).

#### 3.4.2 Effect of PR combined with IMT on PImax in older COPD patients

**FIGURE 4:**
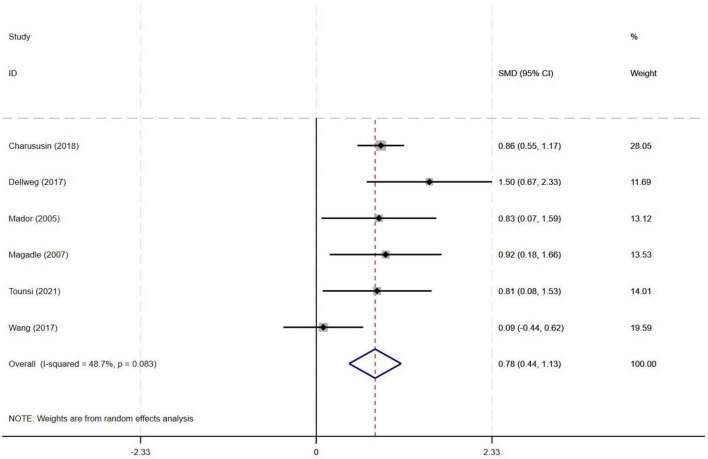
Effect of PR combined with IMT on PImax in older COPD patients.

Among the nine included studies, six evaluated the effects of PR combined with IMT versus PR alone on PImax in older COPD patients. The meta-analysis demonstrated a pooled SMD of 0.78 (95% CI: 0.44–1.13), indicating a statistically significant improvement in PImax with PR combined with IMT compared to PR alone. Heterogeneity among the studies was moderate (I^2^ = 48.7%, *p* = 0.083) (Figure 4).

#### 3.4.3 Effect of PR combined with IMT on FEV_1_ in older COPD patients

**FIGURE 5:**
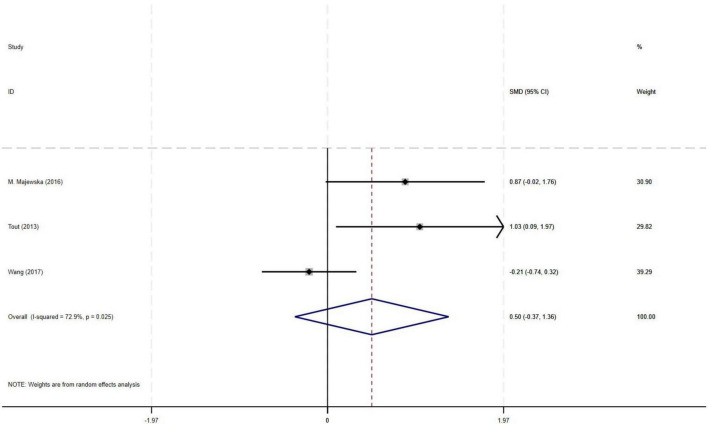
Effect of PR combined with IMT on FEV_1_ in older COPD patients.

The meta-analysis of three RCTs demonstrated a moderate effect size (SMD = 0.50) for improvement in FEV_1_ with PR combined with IMT compared to PR alone in older COPD patients. However, the observed effect did not reach statistical significance, likely attributable to substantial heterogeneity among studies (I^2^ = 72.9%, *p* = 0.025, 95% CI: −0.37–1.36) (Figure 5).

#### 3.4.4 Effect of PR combined with IMT on FEV_1_% in older COPD patients

**FIGURE 6:**
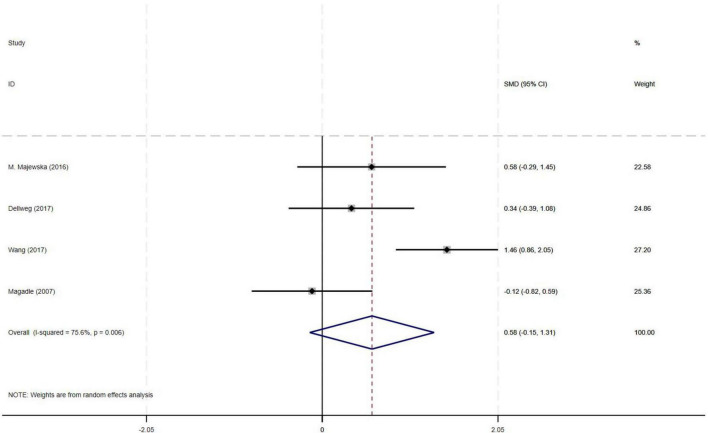
Effect of PR combined with IMT on FEV_1_% in older COPD patients.

The meta-analysis of four RCTs demonstrated a moderate effect size (SMD = 0.58) for improvement in FEV_1_% with PR combined with IMT compared to PR alone in older COPD patients. However, the observed effect did not reach statistical significance, likely attributable to substantial heterogeneity among studies (I^2^ = 75.6%, *p* = 0.006, 95% CI: −0.15–1.31) (Figure 6).

#### 3.4.5 Effect of PR combined with IMT on SGRQ in older COPD patients

**FIGURE 7:**
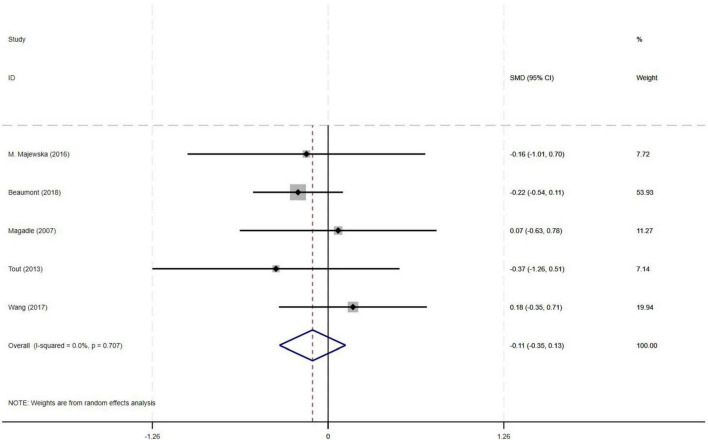
Effect of PR combined with IMT on SGRQ in older COPD patients.

Among the nine included studies, five compared the effects of PR combined with IMT versus PR alone on SGRQ in older COPD patients. The meta-analysis revealed a pooled SMD of −0.11 (95% CI: −0.35–0.13) for SGRQ scores, indicating no statistically significant difference between PR combined with IMT and PR alone. There was no heterogeneity across studies (I^2^ = 0%, *p* = 0.707) (Figure 7).

### 3.5 Subgroup analyses

Subgroup analyses were performed for 6MWD and PImax. Variables related to exercise intervention, including intervention duration (weeks), frequency, session duration, and weekly cumulative duration, may influence outcomes in older COPD patients. Therefore, this study conducted subgroup analyses to investigate whether these factors contribute to heterogeneity in 6MWD and PImax improvements.

Subgroup analyses indicated that PR combined with IMT significantly improved 6MWD in subgroups with intervention durations < 6 weeks and frequencies ≥ 5 sessions/week, demonstrating a moderate effect size (SMD = 0.532). However, the reliability of these findings was limited by substantial heterogeneity (I^2^ = 86.1%). In contrast, subgroups with intervention durations ≥ 6 weeks, frequencies of 3 to <5 sessions/week, session durations ≥ 30 min, or total weekly durations ≥ 180 min showed minimal clinical differences (SMD < 0.2), suggesting no meaningful advantage of PR combined with IMT over PR alone for 6MWD. Collectively, the current evidence does not support a significant improvement in 6MWD with PR combined with IMT, indicating that exercise intervention protocols had no effect on the outcomes (Supplementary Figure 1). Overall, the current evidence is not sufficient to support a generalized improvement in 6MWD in older COPD patients with PR combined with IMT.

Subgroup analyses of PImax showed significant improvements with PR combined with IMT in subgroups with intervention durations ≥ 12 weeks (SMD = 0.866, 95% CI: 0.579–1.153; I^2^ = 0%) and in subgroups with session duration ≥ 60 min or weekly cumulative duration ≥ 180 min (SMD = 0.922, 95% CI: 0.666–1.177; I^2^ = 0%), with no heterogeneity (*p* > 0.05). Subgroups with a duration of 6–12 weeks (SMD = 0.517, 95% CI: 0–1.034; I^2^ = 45.2%) or a frequency of 3–5 sessions/week (SMD = 0.686, 95% CI: 0.36–1.012; I^2^ = 39%) exhibited positive trends but moderate heterogeneity, suggesting potential influences from protocol variations. Subgroups with single exercise sessions of 30–60 min or weekly cumulative duration < 180 min (SMD = 0.406, 95% CI: −0.289–1.10, I^2^ = 59.1%, *p* > 0.05) showed moderate heterogeneity (I^2^ = 59.1%), indicating an unclear direction of effects (Table 3 and Supplementary Figure 2).

**TABLE 3:** PImax subgroup analysis by intervention duration.

Measurements	N	SMD	95% CI	Heterogeneity test results	
				I^2^	*p*
**Duration (weeks)**					
<6 weeks	1	1.499	(0.669, 2.33)	–	–
6 weeks ≤ and <12 weeks	3	0.517	(−0, 1.034)	45.2%	0.161
≥12 weeks*	2	0.866	(0.579, 1.153)	0%	0.877
**Frequency (sessions/week)**					
3≤ and <5 sessions/week	5	0.686	(0.36, 1.012)	39.00%	0.161
≥5 sessions/week	1	1.499	(0.669, 2.33)	–	–
**Session duration (min)**					
30≤ and <60 min	2	0.406	(−0.289, 1.1)	59.10%	0.118
≥60 min*	4	0.922	(0.666, 1.177)	0.00%	0.556
**Weekly cumulative duration (min)**					
<180 min	2	0.406	(−0.289, 1.1)	59.10%	0.118
≥180 min*	4	0.922	(0.666, 1.177)	0.00%	0.556

*Denotes the optimal parameters.

### 3.6 Sensitivity analysis

Sensitivity analysis showed that excluding any single study did not alter the overall effect direction or the consistency of 95% CIs for PR combined with IMT on 6MWD (Supplementary Figure 3) and PImax (Supplementary Figure 4) in older COPD patients. Pooled estimates remained robust, with no significant changes in effect magnitude or significance, confirming the stability of the meta-analysis.

### 3.7 Publication bias

Publication bias was evaluated by examining funnel plots. Visual inspection of the funnel plots for 6MWD (Supplementary Figure 5) and PImax (Supplementary Figure 6) revealed no evidence of asymmetry. Egger’s test showed that small-sample studies did not significantly influence the overall results (6MWD: *p* = 0.051; PImax: *p* = 0.778).

### 3.8 Meta regression

Meta-regression analysis was performed on key intervention parameters including intervention duration, frequency, session duration, and weekly cumulative duration, as well as participant disease staging, with respect to 6MWD (Supplementary Figure 7) and PImax (Supplementary Figure 8) as outcomes. No significant associations were detected between intervention duration (6MWD: *p* = 0.702; PImax: *p* = 0.942), frequency (6MWD: *p* = 0.544; PImax: *p* = 0.547), session duration (6MWD: *p* = 0.497; PImax: *p* = 0.433), or weekly cumulative duration (6MWD: *p* = 0.844; PImax: omitted due to collinearity) and the measured outcomes.

### 3.9 Certainty of evidence

In the comparison of PR combined with IMT versus PR alone in older COPD patients, the GRADE assessment showed that the evidence certainty for the outcomes was low (PImax, 6MWD, SGRQ, FEV1%) or very low (FEV1) (Supplementary Figure 9). The main limitations included imprecision (characterized by small sample sizes and wide confidence intervals) and severe heterogeneity for certain outcomes (e.g., FEV1%, FEV1).

## 4 Discussion

This meta-analysis included 9 randomized controlled trials (582 older COPD patients) to investigate the effects of PR combined with IMT versus PR alone on lung function and exercise endurance. Primary outcomes showed no significant differences between PR combined with IMT and PR alone in improving 6MWD (SMD = 0.15, 95% CI: −0.11–0.42) or SGRQ scores (SMD = −0.19, 95% CI: −0.38–0.01). Although PR combined with IMT demonstrated a moderate effect size for maximal inspiratory pressure (PImax; SMD = 0.78, 95% CI: 0.44–1.13), the evidence was downgraded due to moderate heterogeneity (I^2^ = 48.7%). For lung function, FEV_1_ and FEV_1_% predicted values trended positively (SMD = 0.50) but did not reach statistical significance, attributable to high heterogeneity (I^2^ = 72.9%) and a CI crossing the null effect (95% CI: −0.37–1.36).

While prior studies reported PR combined with IMT improved FEV_1_ in COPD patients, our meta-analysis found FEV_1_ trended positively (SMD = 0.50) but did not reach statistical significance (95% CI: −0.37–1.36, I^2^ = 72.9%) (15, 16). This highlights potential heterogeneity in patient populations, notably driven by this study’s unique inclusion of patients with GOLD stage II–IV and older age. Bodduluri et al. analyzed airway trees in 7,641 participants from the COPD Gene cohort and found that T-Slope–a quantitative measure of airway lumen narrowing–decreased progressively with GOLD stage severity (Jonckheere-Terpstra *p* = 0.04) (35). Critically, T-Slope was independently associated with both FEV_1_ [β = 0.13 (95% CI: 0.10–0.15) L; *p* < 0.001] and annual FEV_1_ decline rate [β = −4.50 (95% CI: −7.32 to −1.67) mL⋅year^–1^; *p* = 0.001] (35), confirming irreversible structural damage (e.g., airway fibrosis, alveolar destruction) as the core driver of airway obstruction (36). This structural resistance may dissociate gains in respiratory muscle strength (PImax) from functional outcomes (6MWD/SGRQ) through dual physiological constraints. In moderate-severe COPD (GOLD II–IV), irreversible parenchymal destruction such as airway fibrosis and alveolar loss reduces pulmonary elastic recoil, compromising compensatory capacity for increased physiological dead space and dynamic hyperinflation despite improved respiratory muscle efficiency (37, 38). Concurrently in older patients, elevated respiratory muscle workload heightens oxygen consumption, diverting metabolic resources from locomotor muscles via the “respiratory metaboreflex” phenomenon–an effect exacerbated in advanced GOLD stages due to elevated baseline ventilatory demands (39, 40). This constraint may be partially mitigated by targeted inspiratory muscle warm-up prior to training, which has been shown to significantly enhance inspiratory strength and efficiency, thereby reducing respiratory oxygen demand and improving functional exercise capacity in moderate-to-severe COPD patients with inspiratory muscle weakness (41). This aligns with the pathological characteristics of older patients and those with high GOLD stages in our study: their profound loss of pulmonary elastic recoil–exacerbated by age-related respiratory muscle degeneration–limits the structural benefits of PR combined with IMT (e.g., airway remodeling reversal). This finding aligns with the pathological characteristics of elderly patients and those with advanced GOLD stages: their profound loss of pulmonary elastic recoil–exacerbated by age-related respiratory muscle degeneration–limits the structural benefits of PR combined with IMT (e.g., airway remodeling). PR and IMT predominantly promote functional adaptations (e.g., enhanced muscle efficiency), which cannot reverse established parenchymal damage (42). Notably, our meta-analysis included fewer studies reporting FEV_1_/FEV_1_% outcomes, and substantial methodological heterogeneity across trials (e.g., varying training protocols) likely contributed to inconsistent cumulative effects of interventions. Thus, while PR combined with IMT showed moderate PImax benefits (SMD = 0.78), the lack of significant FEV_1_ gains and high heterogeneity (I^2^ = 72.9%) hinder definitive conclusions on respiratory mechanics optimization.

Current evidence suggests that standalone respiratory training improves PImax in COPD patients (43–45). However, PR combined with IMT does not confer additional PImax benefits over PR alone (15). Ammous et al. reported that PR combined with IMT increased PImax by 11.46 cmH_2_O (95% CI: 7.42–15.50) versus PR alone, but this fell short of the minimal clinical important difference (MCID) of 17.2 cmH_2_O (16). This discrepancy with our findings may stem from our study’s strict inclusion of patients aged ≥ 55 years, a population vulnerable to age-related respiratory muscle decline and sarcopenia (46, 47). Ammous’ subgroup analysis found no differences between PR combined with IMT and PR alone across duration, frequency, session duration, and weekly cumulative duration [short-term (< 4 weeks), mid-term (4–7 weeks), long-term (≥8 weeks)] (16). In contrast, our PImax subgroup analysis showed that PR combined with IMT significantly and robustly improved PImax versus PR alone when intervention duration was ≥ 12 weeks (SMD = 0.866, 95% CI: 0.579–1.153), session duration ≥ 60 min (SMD = 0.922, 95% CI: 0.666–1.177), or weekly cumulative duration ≥ 180 min (SMD = 0.922, 95% CI: 0.666–1.177). Notably, we found no significant associations between PImax and duration, frequency, weekly cumulative duration, or total intervention time (*p* > 0.05). These divergent meta-analytic results likely reflect heterogeneity in subgroup definitions based on duration, frequency, session duration, and weekly cumulative duration. Given the substantial variability in IMT protocols across included studies–particularly regarding session duration and weekly cumulative duration–no definitive recommendations for COPD patients based on these parameters can be made at this time. Future research, including *a priori* stratification by baseline PImax to address its influence on treatment response, is needed to determine optimal PR combined with IMT parameters for PImax improvement.

Although our study observed a positive trend for PR combined with IMT on FEV_1_ and FEV_1_%, the small number of included studies and high heterogeneity undermined the reliability of these results. Standalone PR and IMT both improve exercise capacity and lung function in COPD patients (9, 16). Notably, clinical significance for such improvements requires meeting MCID thresholds (e.g., 25–30 m for 6MWT; 4 units for SGRQ).This observation may be explained by the intrinsic mechanisms of PR. Güneş et al. demonstrated that 6 weeks of PR (3 sessions/week) significantly increased diaphragm thickness in COPD patients (48). The endurance training components of PR (e.g., walking, cycling) indirectly activate respiratory muscles through increased ventilatory demand, potentially blunting the specific benefits of IMT. Notably, the meta-regression revealed no significant associations between intervention parameters (duration, frequency, session duration, weekly cumulative duration) and outcomes (6MWD/PImax)–contrasting with subgroup analyses indicating that these factors heterogeneously influenced treatment efficacy. This apparent discrepancy likely arises because subgroup analyses identified threshold-dependent effects (e.g., significant PImax improvements only at intervention durations ≥ 12 weeks or weekly cumulative durations ≥ 180 min), while meta-regression modeled parameters as continuous variables, potentially obscuring non-linear relationships. Furthermore, substantial heterogeneity in intervention protocols (evident in the I^2^ = 86.1% for 6MWD in < 6-week subgroups) may have compromised statistical power to detect meta-regression associations (49). Lötters et al. (50) recommended IMT as an adjunct for patients with severe baseline inspiratory muscle weakness (PImax < 60% predicted), whereas the lack of baseline stratification in our study may have diluted treatment effects due to heterogeneous patient populations. This underscores the need for future studies to perform stratified analyses based on baseline respiratory muscle function to establish optimal IMT eligibility criteria.

This study has several limitations that warrant attention. The incomplete reporting of baseline FEV_1_ across studies prevented robust stratification by COPD severity (GOLD stages), contributing to unmeasured heterogeneity in subgroup analyses. The absence of baseline PImax stratification likely obscured differential effects in clinically distinct subgroups (e.g., severe vs. moderate respiratory muscle weakness). Predominant short-term follow-up durations (median ≤ 12 weeks in most trials) preclude conclusions about long-term sustainability (e.g., ≥1 year follow-up data). Heterogeneity in IMT protocols–session duration, frequency, cumulative weekly duration–complicated synthesis of optimal intervention parameters and dose-response relationships. Furthermore, exclusion of non-English publications and limited non-Western representation may restrict applicability to diverse healthcare contexts, as non-English studies (e.g., Chinese) may report distinct intervention efficacies. Finally, the exclusive focus on RCT designs may overlook implementation evidence from real-world settings.

## 5 Conclusion

Pulmonary rehabilitation combined with IMT improved PImax in older COPD patients compared to PR alone, but no advantages of PR combined with IMT were observed for exercise capacity (e.g., 6MWD) or lung function (e.g., 6MWD). For clinicians aiming to enhance respiratory muscle strength in older COPD patients, this meta-analysis suggests that PR combined with IMT lasting ≥ 12 weeks, with individual sessions exceeding 60 min and total weekly intervention time reaching 180 min, may be beneficial. However, these recommendations are based on low-to-very-low quality evidence, and the optimal protocol requires further validation through large-scale, standardized clinical trials.

## Data Availability

The original contributions presented in this study are included in this article/Supplementary material, further inquiries can be directed to the corresponding authors.
