# Supplementary material for: Effects of pulmonary rehabilitation combined with inspiratory muscle training on lung function and exercise capacity in older patients with COPD: a systematic review and meta-analysis
Source: Front Med (Lausanne). 2025 Jul 9;12:1621375. doi: 10.3389/fmed.2025.1621375 (PMC12285735; doi:10.3389/fmed.2025.1621375)

Supplementary Material

Supplementary Table 1

Search terms include: Exercise, Aerobic Exercise, Respiratory Muscle Training, Pulmonary rehabilitation, Aged, Chronic Obstructive Pulmonary Disease, COPD, Exercise capacity, Exercise tolerance, pulmonary function, and Randomized controlled trial, etc. The specific search strategy is as follows：

Web of Science：

1: TS= (Exercise OR Exercise* OR Physical Exercise* OR Aerobic Exercise* OR Isometric

2: TS= (Breathing Exercises OR Breathing Exercise* OR Respiratory Muscle OR breathing

training OR pulmonary rehabilitation)

3: TS= (elderly OR aged)

4: TS= (Pulmonary Disease, Chronic Obstructive OR Chronic Obstructive Pulmonary Disease* OR COPD OR Chronic Obstructive Lung Disease OR COAD OR Chronic Obstructive Airway Disease OR Airflow Obstruction, Chronic OR Airflow Obstructions, Chronic OR Chronic Airflow

5: TS= (Exercise Tolerance OR Tolerance, Exercise OR exercise capacity)

6: TS= (respiratory function OR pulmonary function OR lung function)

7: #5 AND #6

8: TS= (randomized controlled trial OR randomized OR placebo)

9: #1 AND #2 AND #3 AND #4 AND #7 AND #8

Embase：

| #19 | #3 AND #6 AND #9 AND #12 AND #17 AND #18 |
| --- | --- |
| #18 | 'randomized controlled trial':ab,ti OR 'randomized':ab,ti OR 'placebo':ab,ti |
| #17 | #13 OR #14 OR #15 OR #16 |
| #16 | 'respiratory function':ab,ti OR 'pulmonary function':ab,ti OR 'lung function':ab,ti |
| #15 | 'respiratory function'/exp OR 'respiratory function' |
| #14 | 'exercise tolerance':ab,ti OR 'tolerance,  exercise':ab,ti OR 'exercise capacity':ab,ti |
| #13 | 'exercise tolerance'/exp OR 'exercise tolerance' |
| #12 | #10 OR #11 |
| #11 | 'pulmonary disease, chronic obstructive':ab,ti OR 'chronic obstructive pulmonary disease*':ab,ti OR 'copd':ab,ti OR 'chronic obstructive lung  disease':ab,ti OR 'coad':ab,ti OR 'chronic  obstructive airway disease':ab,ti OR 'airflow obstruction, chronic':ab,ti OR 'airflow  obstructions, chronic':ab,ti OR 'chronic airflow obstruction*':ab,ti |
| #10 | 'chronic obstructive lung disease'/exp |
| #9 | #7 OR #8 |
| #8 | 'elderly'/exp OR elderly OR aged:ab,ti |
| #7 | 'aged'/exp OR 'aged' |
| #6 | #4 OR #5 |
| #5 | 'breathing exercises':ab,ti OR 'breathing  exercise*':ab,ti OR 'respiratory muscle':ab,ti OR 'breathing training':ab,ti OR 'pulmonary  rehabilitation':ab,ti |
| #4 | 'breathing exercise'/exp OR 'breathing exercise' |
| #3 | #1 OR #2 |
| #2 | 'exercise':ab,ti OR 'exercise*':ab,ti OR 'physical exercise*':ab,ti OR 'aerobic exercise*':ab,ti OR  'isometric exercise*':ab,ti |
| #1 | 'exercise'/exp |

Cochrane：

#1 MeSHdescriptor: Exercise/exp

#2 (Exercise.ti,ab OR Exercise*.ti,ab OR Physical Exercise*.ti,ab OR Aerobic Exercise*.ti,ab OR Isometric Exercise*.ti,ab)

#3 #1 OR #2

#4 MeSHdescriptor: "Breathing Exercises"/exp

#5 (Breathing Exercises.ti,ab OR Breathing Exercise*.ti,ab OR Respiratory Muscle.ti,ab OR breathing training.ti,ab OR pulmonary rehabilitation.ti,ab)

#6 #4 OR #5

#7 MeSHdescriptor: Aged/exp

#8 (elderly.ti,ab OR aged.ti,ab)

#9 #7 OR #8

#10 MeSHdescriptor: "Pulmonary Disease, Chronic Obstructive"/exp

#11 (Pulmonary Disease, Chronic Obstructive.ti,ab OR "Chronic Obstructive Pulmonary Disease*".ti,ab OR COPD.ti,ab OR "Chronic Obstructive Lung Disease".ti,ab OR COAD.ti,ab OR "Chronic Obstructive Airway Disease".ti,ab OR "Airflow Obstruction, Chronic".ti,ab OR "Airflow Obstructions, Chronic".ti,ab OR "Chronic Airflow Obstruction*".ti,ab)

#12 #10 OR #11

#13 MeSHdescriptor: "Exercise Tolerance"/exp

#14 (Exercise Tolerance.ti,ab OR "Tolerance, Exercise".ti,ab OR "exercise capacity".ti,ab)

#15 (respiratory function.ti,ab OR "pulmonary function".ti,ab OR "lung function".ti,ab)

#16 #13 OR #14 OR #15

#17 (randomized controlled trial.ti,ab OR randomized.ti,ab OR placebo.ti,ab)

#18 #3 AND #6 AND #9 AND #12 AND #16 AND #17

Pubmed

(((((("Exercise"[Mesh]) OR ((((Exercise*[Title/Abstract]) OR (Physical Exercise*[Title/Abstract])) OR (Aerobic Exercise*[Title/Abstract])) OR (Isometric Exercise*[Title/Abstract]))) AND (("Breathing Exercises"[Mesh]) OR ((((Breathing Exercise*[Title/Abstract]) OR (Respiratory Muscle Training[Title/Abstract])) OR (breathing training[Title/Abstract])) OR (pulmonary rehabilitation[Title/Abstract])))) AND (("Aged"[Mesh]) OR ((Aged[Title/Abstract]) OR (Elderly[Title/Abstract])))) AND (("Pulmonary Disease, Chronic Obstructive"[Mesh]) OR (((((((((Pulmonary Disease, Chronic Obstructive[Title/Abstract]) OR (Chronic Obstructive Pulmonary Disease*[Title/Abstract])) OR (COPD[Title/Abstract])) OR (Chronic Obstructive Lung Disease[Title/Abstract])) OR (COAD[Title/Abstract])) OR (Chronic Obstructive Airway Disease[Title/Abstract])) OR (Airflow Obstruction, Chronic[Title/Abstract])) OR (Airflow Obstructions, Chronic[Title/Abstract])) OR (Chronic Airflow Obstruction*[Title/Abstract])))) AND ((((("Exercise Tolerance"[Mesh]) OR (((Exercise Tolerance[Title/Abstract]) OR (Tolerance, Exercise[Title/Abstract])) OR (exercise capacity[Title/Abstract]))) ("Exercise Tolerance"[Mesh])) OR (((Exercise Tolerance[Title/Abstract]) OR (Tolerance, Exercise[Title/Abstract])) OR (exercise capacity[Title/Abstract]))) OR (((respiratory function[Title/Abstract]) OR (pulmonary function[Title/Abstract])) OR (lung function[Title/Abstract])))) AND (randomized controlled trial[Publication Type] OR randomized[Title/Abstract] OR placebo[Title/Abstract])

Supplementary Figure 1


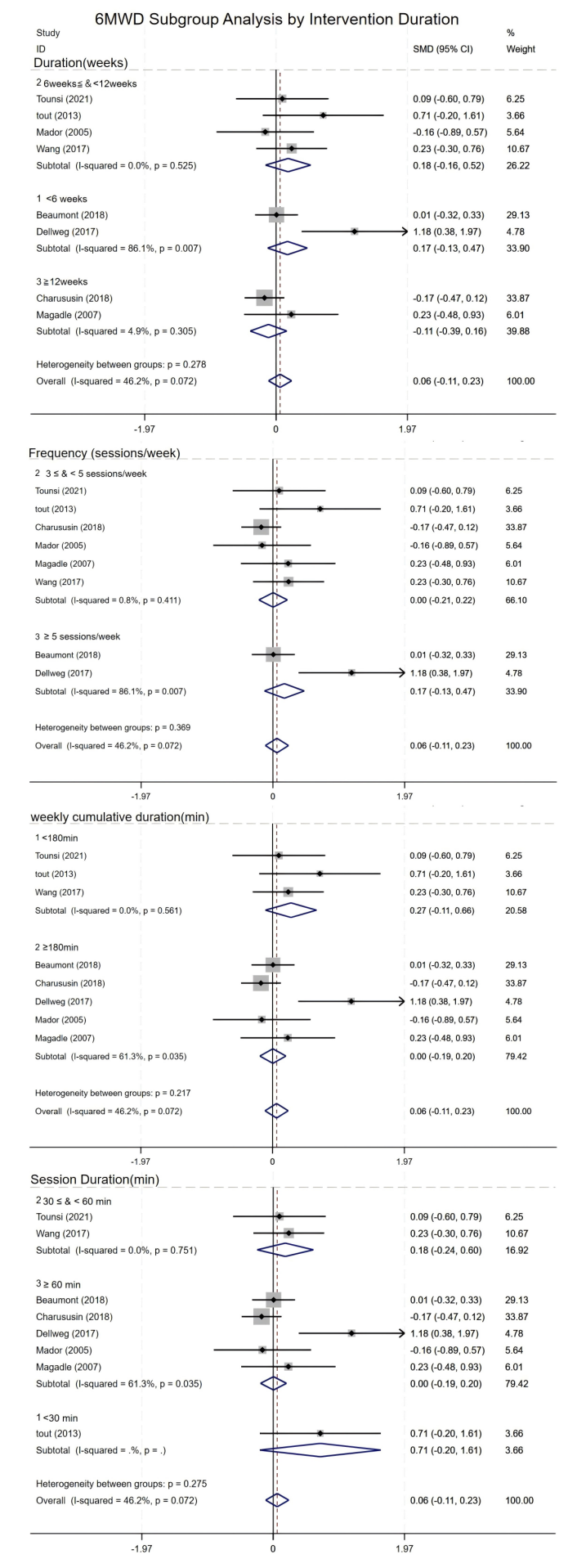


Supplementary Figure 1 6MWD Subgroup Analysis by Intervention Duration

Supplementary Figure 2


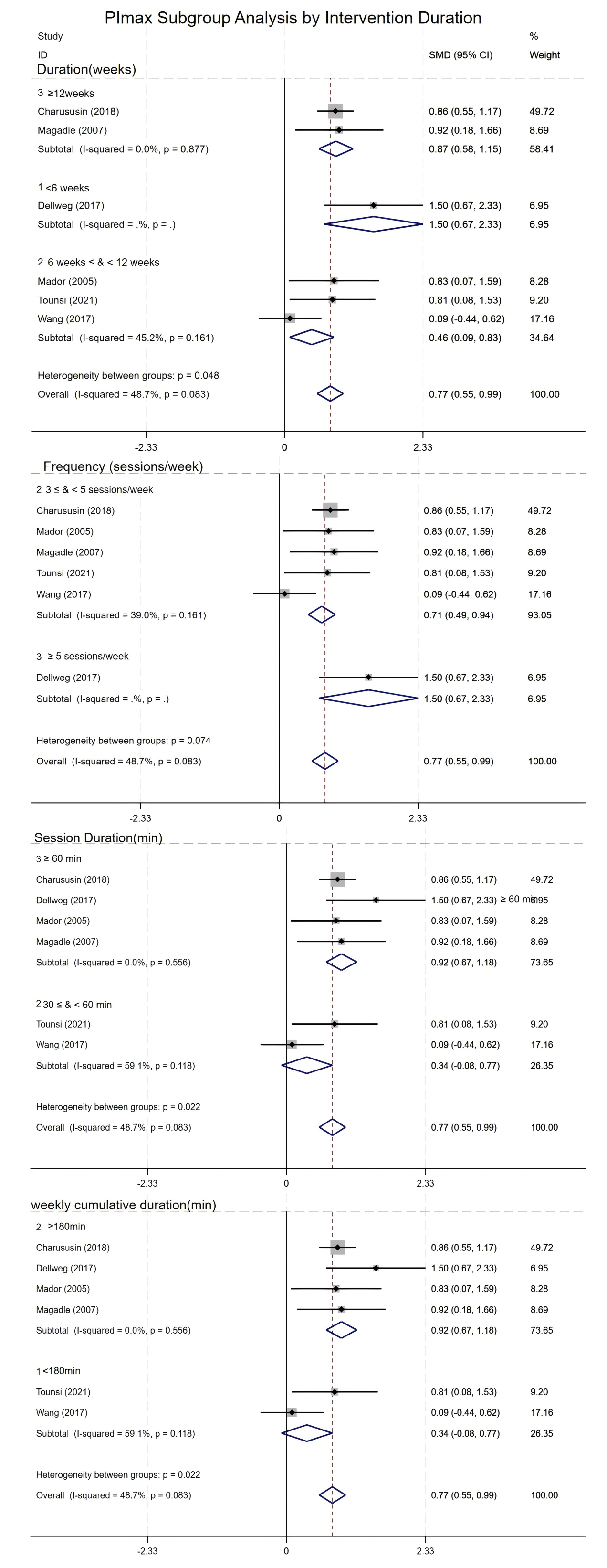


Supplementary Figure 2 PImax Subgroup Analysis by Intervention Duration

Supplementary Figure 3


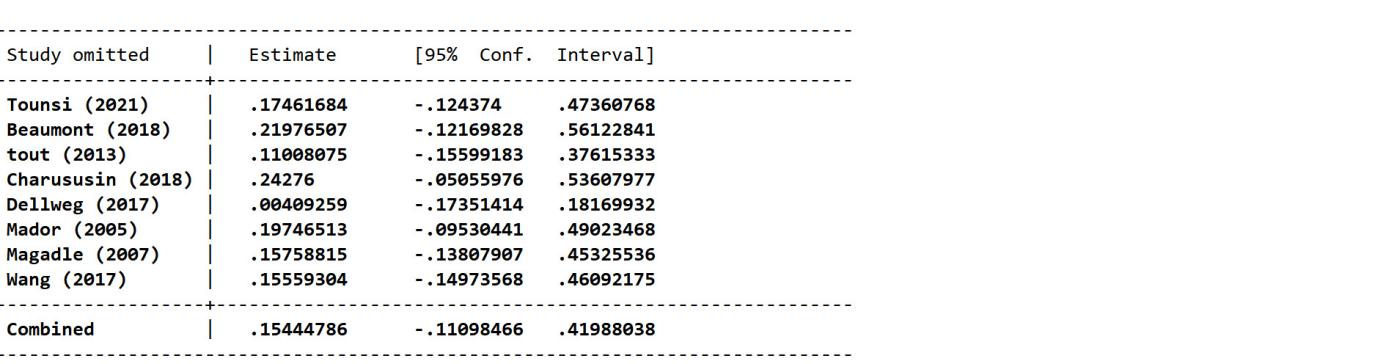


Supplementary Figure 3. Sensitivity Analysis of 6MWD

Supplementary Figure 4


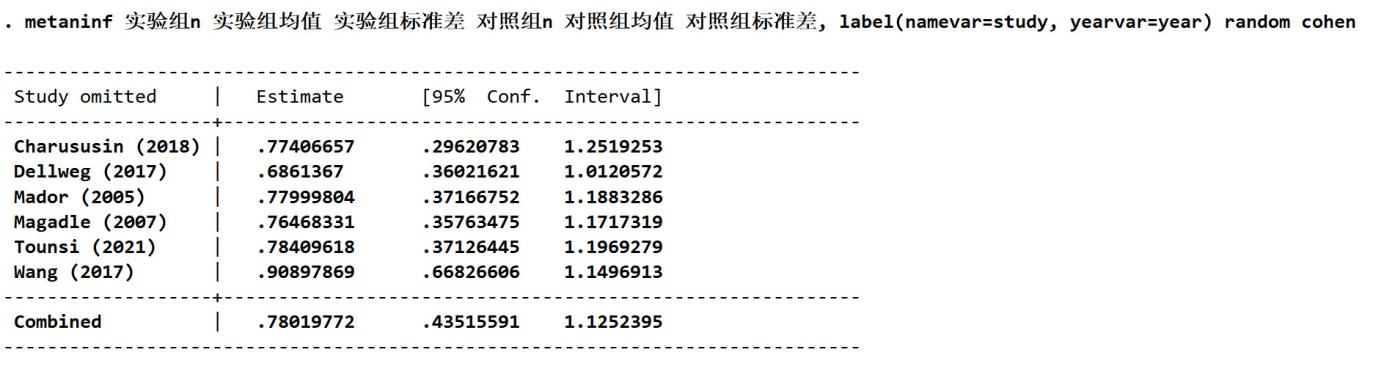


Supplementary Figure 4. Sensitivity Analysis of PImax

Supplementary Figure 5


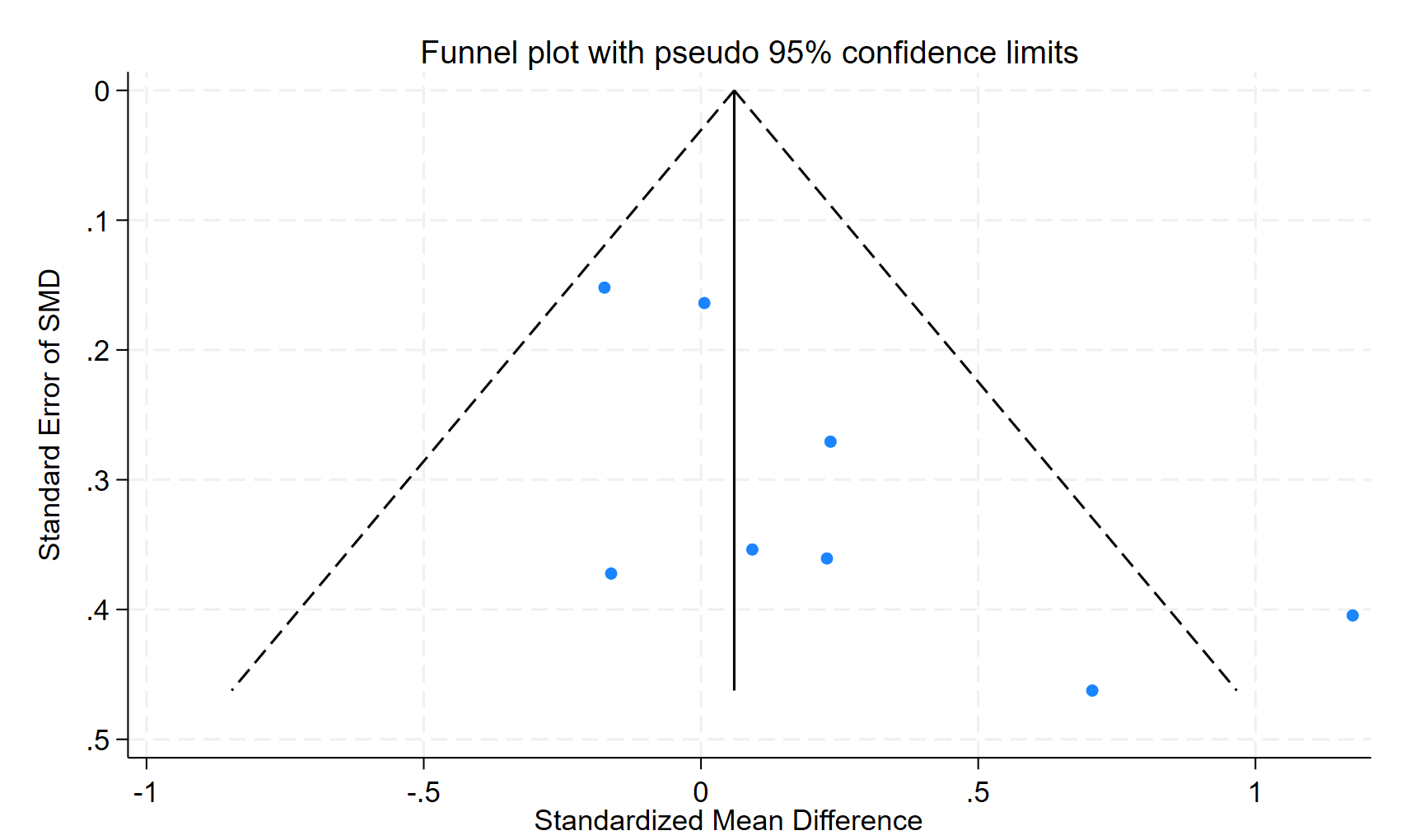


Supplementary Figure 5 Publication Bias of 6MWD

Supplementary Figure 6


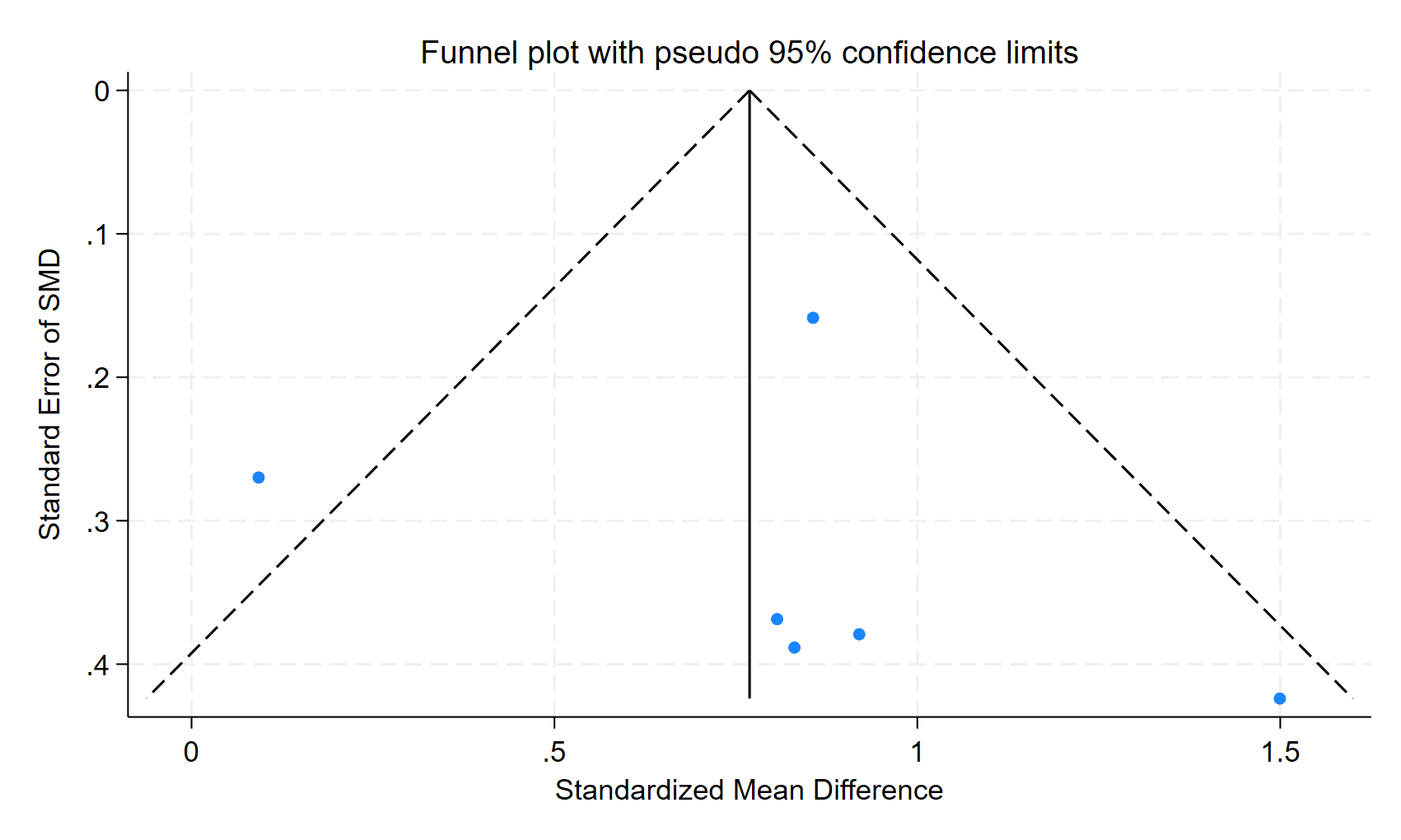


Supplementary Figure 6 Publication Bias of PImax

Supplementary Figure 7


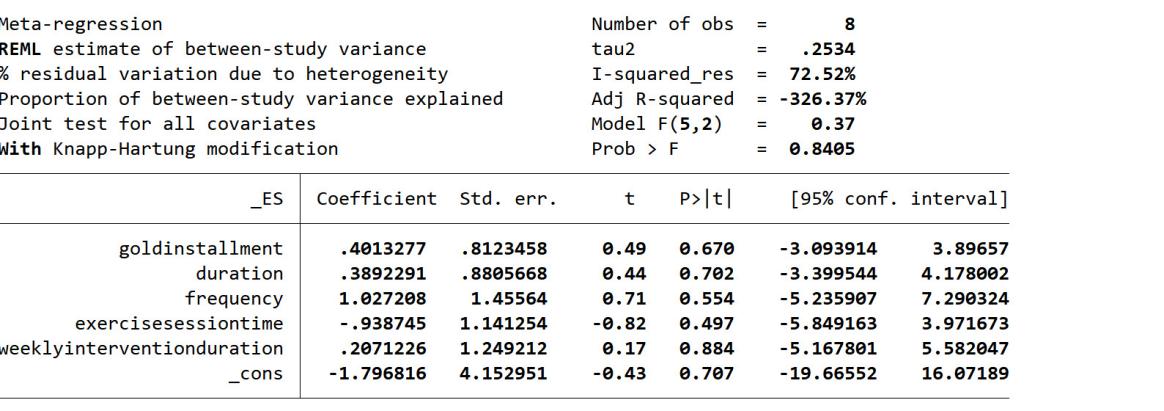


Supplementary Figure 7. Meta-regression analysis of 6WMD

Supplementary Figure 8


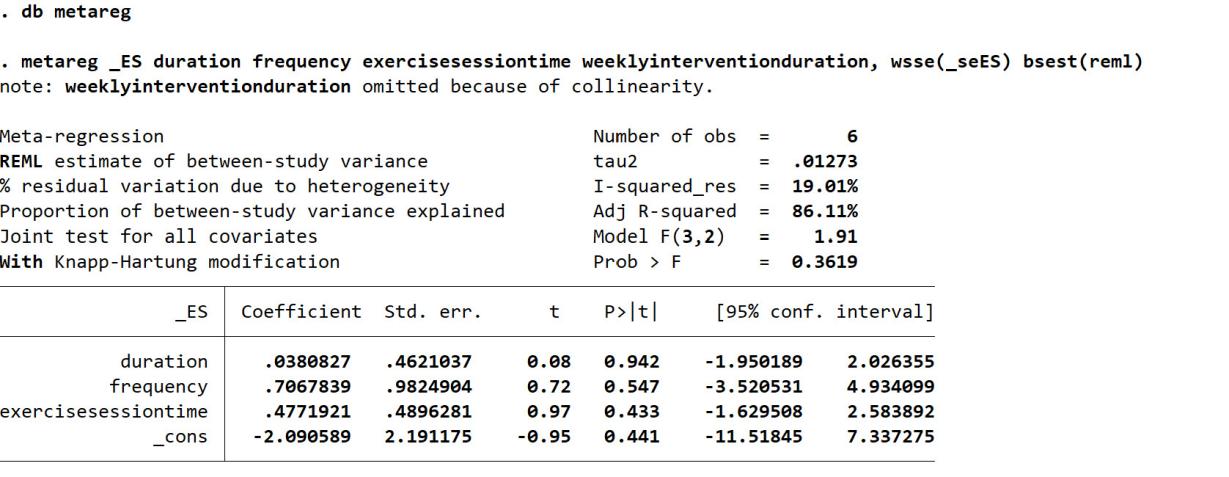


Supplementary Figure 8. Meta-regression analysis of 6WMD

Supplementary Figure 9


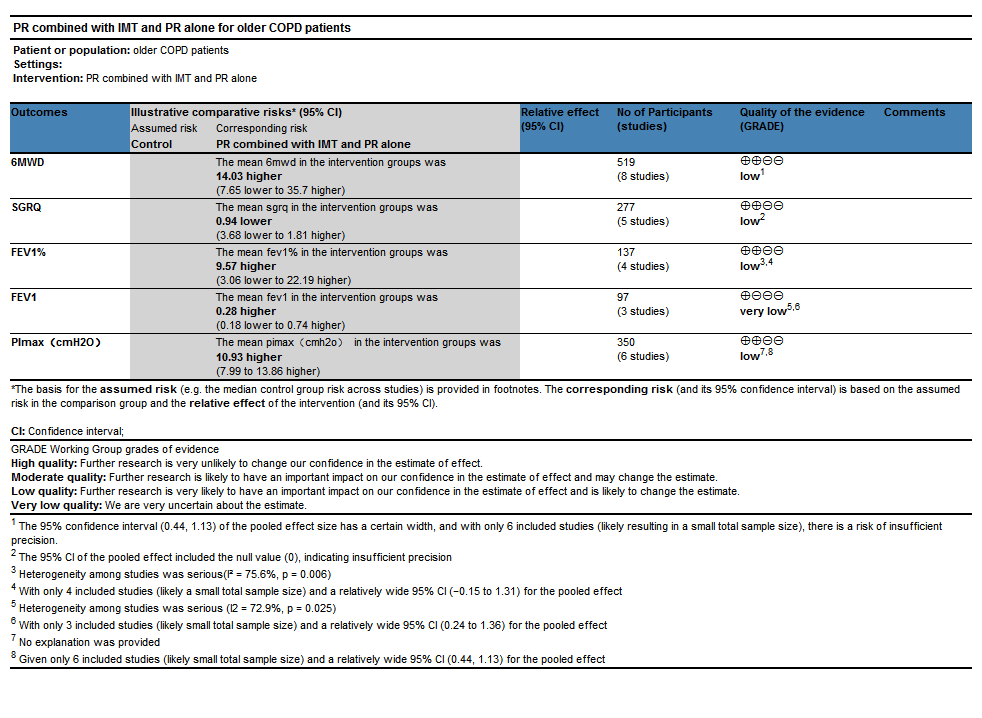

Supplement: Supplementary file 1 [file Supplementary_file_1.docx]
